# Infections in mechanical circulatory support

**DOI:** 10.3389/fcvm.2026.1910836

**Published:** 2026-08-05

**Authors:** Pallabi Shrestha, Amitoj Singh, D. Alexander Perry, Tushar Acharya, Muhammad Ijaz Pervez, Jessica DeAngelo De La Rosa, Jacqueline R. Finger, Toshinobu Kazui, Deepak Acharya

**Affiliations:** 1 Division of Cardiology, University of Arizona, Tucson, AZ, United States; 2 Division of Infectious Diseases, University of Arizona, Tucson; 3 Division of Cardiothoracic Pharmacy, Banner University Medical Center, Tucson, AZ, United States; 4 Division of Cardiothoracic Surgery, Banner University Medical Center, Tucson, AZ, United States

**Keywords:** advanced heart failure, driveline, heart transplant, infection, left ventricular assist device (LVAD)

## Abstract

Mechanical circulatory support (MCS) devices represent a major advance in the management of advanced heart failure and cardiogenic shock. Despite significant improvements in device technology and patient outcomes, infection remains the most common adverse event, affecting approximately 37% of patients and contributing substantially to morbidity and mortality. This state-of-the-art, multidisciplinary review comprehensively examines infections in patients who require durable or temporary mechanical circulatory support devices with contributions from experts in advanced heart failure, cardiovascular imaging, infectious diseases, cardiothoracic surgery, and pharmacology. We review the underlying pathophysiology of MCS-related infections, examine current preventative, diagnostic and management strategies, outline established best practices, discuss emerging technologies and therapies, and identify existing knowledge gaps and areas for future investigation.

## Introduction

Mechanical circulatory support (MCS) devices provide hemodynamic support in advanced heart failure and cardiogenic shock. Conditions once considered uniformly fatal can now be managed with short- and long-term MCS strategies that bridge patients to myocardial recovery or heart transplantation or provide durable destination therapy. Technological advances and improved outcomes have driven widespread adoption of temporary MCS (tMCS) devices, including micro-axial pumps and extracorporeal membrane oxygenation, as well as durable left ventricular assist devices (LVADs). Despite reductions in complications such as stroke, pump thrombosis, and gastrointestinal bleeding, device-related infections remain common and are associated with substantial morbidity and mortality, representing a persistent limitation of contemporary MCS therapy.

Evidence guiding the prevention and management of MCS-associated infections remains limited, particularly for temporary devices, and current practice is largely based on expert consensus, and a significant proportion of the underlying evidence base is derived from single-center, retrospective, observational, or heterogeneous studies (1). Existing consensus statements and guidelines on infection in MCS originate from disparate time periods—some nearly a decade old—and do not reflect recent advances. While these documents address specific domains such as definitions, prevention, and management, they do not fully integrate these elements into a unified perspective that captures their interrelationships. This review addresses that gap by synthesizing the full spectrum of relevant concepts into a single, comprehensive manuscript and offering a contemporary, practical framework for the management of MCS infections. Knowledge gaps and future research directions are highlighted. Durable MCS is discussed first, followed by temporary MCS devices.

## Durable mechanical circulatory support

### Epidemiology

**Figure 1:**
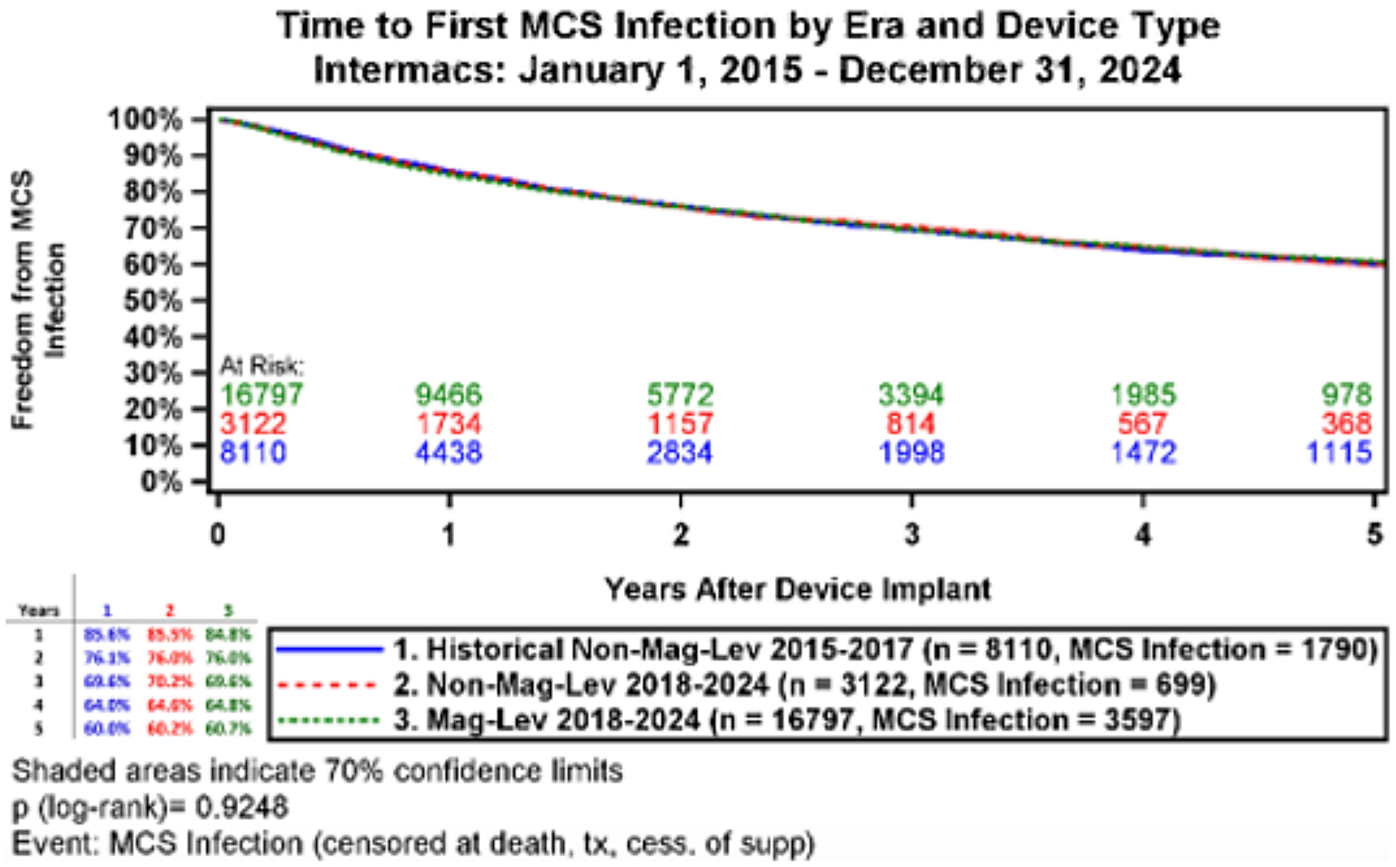
Kaplan–Meier estimates showing freedom from mechanical circulatory support (MCS) device infection for patients with non–magnetically levitated (Mag-Lev) devices in the historical era (2015−2017) and non–Mag-Lev, and Mag-Lev devices in the contemporary era (2018−2024). (cess of supp, cessation of support; Intermacs, Interagency Registry for Mechanically Assisted Circulatory Support; tx, transplant).

Following the advent of cardiopulmonary bypass in the 1950s, early MCS devices emerged in the 1960s to treat post-cardiotomy shock and failure to separate from bypass. Subsequent advances in ventricular assist devices (VADs), total artificial hearts (TAH), and transplantation gradually transformed initially disappointing results into promising therapies. Early paracorporeal and intracorporeal pulsatile VADs from the 1980–1990s were complicated high infection rates (>50%) and mortality (2). Heartmate XVE in transplant-ineligible patients reported 28% LVAD-related infection rate in the device arm (3). Compared with the large pulsatile XVE, the continuous-flow Heartmate II with smaller pocket had lower infection rates (4). However, subsequent platforms (HVAD, Heartmate 3) have not shown further reductions (Table 1) (5–7). In an International Registry for Mechanically Assisted Circulatory Support (IMACS) registry analysis, infection was the most common adverse event (37% of patients). Non-VAD infections such as pneumonia, gastrointestinal, or genitourinary infections accounted for two thirds of overall infections. VAD-specific infections were predominantly in the driveline (82.9%), followed by pocket (12.8%), and pump/cannula (4.3%). The highest rate of infections occurred in the first 3 months of post implant (8). An Interagency Registry for Mechanically Assisted Circulatory Support (INTERMACS) analysis demonstrated no changes in freedom from infection with different devices between 2015 and 2024 (Figure 1) (9). Overall, LVAD-related infections affect nearly one in six patients in the first year and account for 7% of early and 15% of late mortality, while also predisposing to stroke and pump thrombosis (10). The CardioWest TAH trial showed approximately 18% incidence of driveline infection (DI), 7% bloodstream infection, and 5% mediastinal infection. With more contemporary TAH analyses, overall infection rates were highest in the early phase (≤ 3 months after device implant) at 38.8 events per 100 patient months) but remained important in the late phase(> 3 months after device implant) at 12.3 events per 100 patient months), with major infection rates of 10.5% (11).

**Table 1:** Landmark LVAD studies.

Study/Year Published	Device (s)	Infection rates
REMATCH (2001) (3)	Heartmate XVE	Overall LVAD related infection: 28%. Sepsis:0.6 EPPY; local infection 0.39 EPPY, Driveline/pocket infection: 0.41 EPPY, Infection of pump interior/inflow/outflow: 0.23 EPPY
HM II DT (2009) (4)	Heartmate XVE vs. HM II	LVAD-related infection: 0.48 EPPY (HM II) vs. 0.90) XVE (*p* 0.01) Local non-LVAD infection: 0.76 EPPY (HM II) vs.1.33 (XVE) (*p* 0.02) Sepsis: 0.39 EPPY (HM II) vs. 1.11 EPPY (XVE), *p* < 0.01
ENDURANCE (5)	Heartware vs. HM II	Sepsis: 0.20 EPPY (HVAD) vs. 0.16 (HM2), *p* = 0.048 Driveline infection: 0.18 EPPY (HVAD) vs. 0.13 (HM2 II), *p* = 0.30
MOMENTUM 2-year (2019) (6)	HM3vs. HMII	Any major infection: 0.82 EPPY in both groups. (*p* 0.96) Sepsis: 0.13 EPPY in both groups (*p* 0.94) Driveline infection: 0.23 EPPY (HM3) vs. 0.22 (HM2), (*p* 0.6)
MOMENTUM 5-year (2022) (7)	HM3 vs. HMII	Any major infection: 0.515 (HM3), 0.551 (HM II) (*p* 0.94) Sepsis: 0.1 EPPY both groups (*p* 0.96) Driveline infection: 0.15 EPPY both groups (*p* 0.99)

### Microbiology

The microbiology of VAD infections depends on the timing of the infection and whether the infection is associated with the VAD. VAD-specific infections that occur <1 month after implantation are usually in the intraoperative or perioperative period and can include bacteremia and non-VAD infections (CRBSI, UTI, pneumonia, *C.difficile*) (12). Non-VAD infections can be associated with bacteremia and can seed the VAD. However, driveline infections in the early postoperative period are predominantly from Gram-positive bacteria including Staphylococcus aureus and Staphylococcus epidermidis, which colonize the skin^b^.

Late-onset infections are predominantly associated with driveline maintenance and care. While Gram-positive bacteria predominate in the early-onset infections, Gram-negative bacteria predominate in the late-onset infections. Of these, *Pseudomonas aeruginosa* accounts for 25% of driveline infections, but other Gram-negatives*, Klebsiella pneumoniae*, *Acinetobacter baumannii*, *Enterobacter* species and *Serratia* species are also implicated (12).

While bacteria commonly cause driveline infections, fungi are less frequently implicated and are more often associated with deep tissue infections or VAD-related bloodstream infections. Fungal infections occur in <10% but are morbid with up to 25% mortality and challenging treatment. *Candida albicans* account for about 70%, followed by *Candida glabrata* at 10% (13).

### Pathophysiology

**Figure 2:**
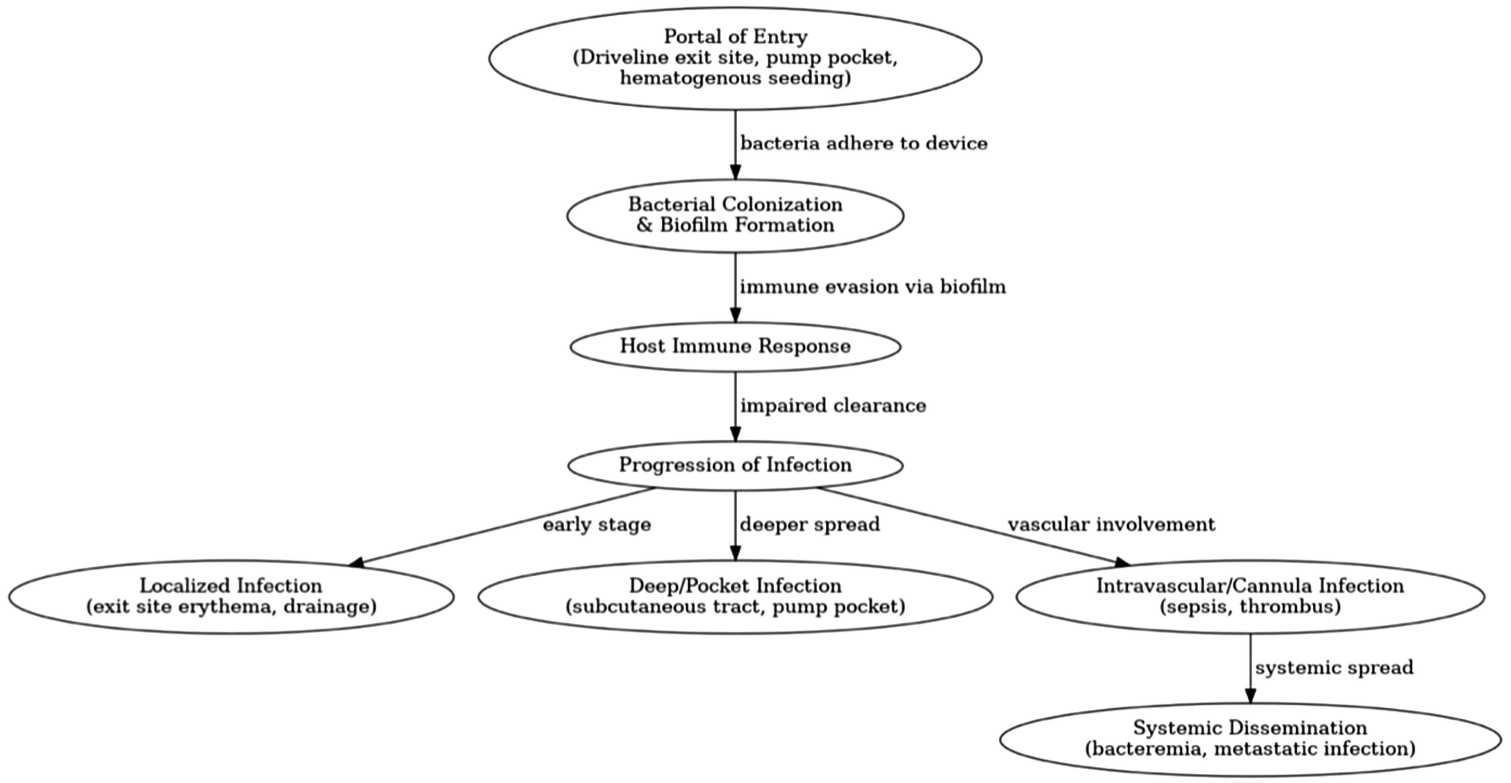
Pathophysiology of device infection, including initiation, colonization, biofilm development, host response, and progression.

Patients with LVADs are highly susceptible to infection from both non–device-related and device-related causes. Non–device-related infections reflect host and care factors, including heart failure–associated immune dysregulation, invasive lines, immobility, malnutrition, and the presence of other intracardiac devices. LVAD-specific infections may involve any device component and arise from perioperative inoculation, ascending contamination along the driveline after skin trauma or breakdown, or hematogenous seeding from remote infections (Figure 2).

Once organisms adhere to device surfaces, eradication is difficult due to poor vascularity, limited immune access, and reduced antibiotic penetration. Inadequate tissue integration and microgaps in the driveline velour facilitate spread, while biofilm formation further impairs antimicrobial efficacy. Biofilms can migrate in the driveline tunnel by expansion or dispersal-seeding, and patients who have clinically superficial DI can have microfilms extending deep into the driveline tunnel. The exit site cultures have high concordance with deeper driveline cultures. Biofilms can have high resistance to conventional antibiotics that were shown to be effective in treating isolated organisms in culture (14, 15).

### Pre-implant, implant, and periprocedural considerations

Because LVAD infections are difficult to treat, prevention should be prioritized. The 2017 ISHLT consensus statement provided a comprehensive overview of perioperative and preventive strategies based on the evidence available at that time. The following section summarizes these recommendations and integrates more recent emerging evidence.

Evaluation of suspected infection in MCS candidates should follow standard clinical assessment, with infectious diseases consultation considered for all patients with suspected or confirmed infection (13). Dental evaluation is recommended when indicated (13). MCS implantation should be deferred until adequate source control is achieved, blood cultures are negative, and clinical signs of infection or sepsis have been resolved (13).

Screening patients for nasal colonization for MRSA and using mupirocin to decontaminate reduces Staphylococcus aureus infections in surgery patients (16, 17). Pre-operative chlorhexidine bath reduces skin bacterial counts but does not reduce post-operative wound infection rates after cardiac surgery (18). Reported antimicrobial allergies with uncertain history should be evaluated, since allergies to penicillin-class antibiotics are overreported and often based on childhood reactions. Removing incorrect antibiotic allergies removes limitations on potential antibiotics used and can be more effective than the substitution in the settings of allergies. Consultation with an allergy specialist can be considered (13).

Perioperative antimicrobial should target Staphylococcus species and can be individualized if a patient is colonized with MRSA or there is high MRSA prevalence based on the antibiogram of the institution. Standard antibiotics, usually cephalosporins (cefazolin or cefuroxime), which cover common skin Gram-positives, can provide some Gram-negative coverage for 24–48 h (19). For the general population, there are no difference in outcomes (LVAD infection, C. diff, all-cause mortality) with narrow spectrum (cefazolin or cefazolin + vancomycin or vancomycin) vs. expanded spectrum antibiotics (cefepime, antifungals) as antibiotic prophylaxis (20). Duration of perioperative antibiotics of upto 48 h is appropriate, as longer courses have not been shown to reduce infection rates and may contribute to antimicrobial resistance (21). Age-specific routine vaccination, nutritional optimization, and glycemic control are recommended in the perioperative period for infection prevention and control (22).

During implantation, standard cardiothoracic surgical implant precautions are taken. There is no consensus on soaking the LVAD components in antibiotic solution prior to implantation, but HM3 manufacturer recommends wrapping the pump and velour portion of the pump cable in antibiotic-soaked laps and massaging the solution into the velour part of the cable (23). Positioning the velour entirely within the subcutaneous tunnel and using a silicone exit interface reduces infection risk (24, 25). The driveline course is often extended in a C configuration to increase the distance from the skin to the pump. A double tunnelling technique, whereby the driveline is placed in the rectus muscle sheath in the umbilical direction and subsequently extended subcutaneously reduced DI at 5 years by 50% in a single center study of 69 patients (26). Anchoring of the driveline to the skin in the first 2–3 weeks post implantation or fixing the driveline exit site at a distant abdominal site to prevent movement at the exit site may also promote healing and reduce infection risk. Negative pressure wound therapy (NPWT) as a prophylactic mechanism for DI prevention has also been evaluated; a 2022 study of 50 patients with NPWT for 7 days post LVAD demonstrated safety but did not show a statistically significant reduction in DI rates at 1 year (32% NPWT vs. 42% control) reduction in DI at 1 year compared to historical controls (27). Conversely, another study where NWPT was applied for 14 days after surgery was associated with a decrease in LVAD infections (11.9% vs. 33.8%) at a median follow-up of 2.06 years in 129 patients (28).

### Driveline management

The 2017 ISHLT consensus statement recommended driveline dressing change with aseptic technique, initially more frequent with daily dressing changes post implantation and then decreased to 1–3 times weekly once healed and without infection. Patients could shower with adequate protective equipment once adequate healing of the exit site occurred (13). A 2021 systematic review of 11 studies with 1602 LVAD patients revealed marked variability in driveline care, without standardized dressing change technique (29). Driveline exit care protocols incorporating chlorhexidine, silver-based dressings, dressing changes 1–2 times a week after healing in the absence of infection, and the use of an anchoring device and dressing kits have been associated with lower infection rates (30). In a single-center study of 153 patients, a fenestrated hydrophilic foam dressing was associated with a significant decrease in DI (31). To standardize dressing care, German and Austrian centers developed the DESTINE protocol (32). More recently, the ASPIRE study evaluated infection-related practice patterns in 30 Heartmate III LVAD centers and correlated these practices to infection rates. The major findings were that there was significant variability in management practices across centers, late DI rate was higher with more frequent (at least daily) dressing changes, and the use of chlorhexidine cleanser with silver-impregnated dressing was associated with lower DI rate than other modalities. There was no correlation with antibiotic prophylaxis, driveline tunneling, and diabetes practice with DI. Best practice recommendations were for chlorhexidine with silver-impregnated dressings, dressing changes every 1–3 days in the first 4 weeks and 3–7 days thereafter, judicious perioperative antibiotic use, standardized staging system and routine photographic driveline imaging, and repeated patient and caregiver training in driveline management and infection recognition (33).

Given the persistently elevated infection rates with current generation devices, driveline management remains an important source of infection prevention and control.

Caregiver involvement is crucial in driveline infection prevention and treatment. Ability to provide consistent dressing change, maintain driveline exit point stability with anchors or other devices, and keeping the exit site dry can help prevent infections. Caregiver surveillance of the exit site and prompt reporting to the LVAD coordinator with any concerns of infection can enable early detection and prompt therapy. The quality and depth of caregiver training across LVAD programs may be heterogeneous. Consistent training, retraining, and support is necessary (34). Chronic driveline infection, particularly with complex dressing change or antibiotic regimens at home can cause caregivers significant psychological burden and may lead to caregiver burnout and increase the risk of dressing nonadherence or delayed reporting of wound changes. LVAD programs must be vigilant in providing needed material and psychological support to caregivers (35).

### Infection definitions

Early LVAD trials used study-specific infection definitions, limiting comparability as LVAD use expanded and multiple trials and registries emerged (36). In 2011, the ISHLT Infectious Diseases Working Group created the first consensus-driven, standard definitions for LVAD infections. Infections were divided into 3 main categories: VAD-specific, VAD-related infections and non-VAD infections. VAD specific infections were further subclassified as proven, probable, and possible infections with microbiological, histologic, radiologic, and clinical criteria for classification (37). These definitions were revised in 2024 to account for the proliferation of tMCS devices and to simplify categories. Infections were categorized into MCS-specific and non-MCS specific. MCS specific infections were divided into complicated and uncomplicated percutaneous lead/DI, complicated and uncomplicated vascular cannulation site infections, device specific bloodstream infections, device endocarditis, and infection of external surface of implantable component (1) (Table 2).

**Table 2:** ISHLT updated definitions 2024.

Classification	Diagnostic criteria
MCS specific infections: infections related to device implantation otherwise not present in absence of such devices.	
1. Percutaneous lead (driveline)	
a. Uncomplicated	-Pain, tenderness, erythema, drainage, induration at driveline site. -Positive drainage culture -Clinical improvement with antibiotics
b. Complicated	Above criteria with any: -Fistulous tract at driveline site -Fluid collection/abscess on imaging with positive culture -Systemic signs and symptoms -Positive blood culture -MDRO or fungal infection -Infection of external surface
2. Vascular cannulation site	
a. Uncomplicated	-Pain, tenderness, erythema, drainage, induration at cannula insertion site. -Positive drainage culture
b. Complicated	Above criteria with any: -Fistulous tract at cannulation site -Fluid collection/abscess on imaging with positive culture -Purulence at cannula/sheath/vascular graft -blood vessel interface -Systemic signs and symptoms -Positive blood culture -MDRO or fungal infection -infection of external surface
3. Device specific bloodstream infection	-Positive blood culture with . driveline/cannula/sheath/graft site infection . cannula tip/sheath tip/graft culture infection . external surface infection . device circuit blood culture positive . persistent positive culture with same organism >72 h apart -Systemic signs and systems
4. Device endocarditis	-Positive peripheral or MCS circuit culture -Radiographic or echo evidence of vegetation or thrombus on intravascular aspect of device component -CVA due to septic emboli -Systemic signs and symptoms
5. Infection of external surface of MCSD	-Tissue or fluid culture positive surrounding external surface of MCS or its components -Systemic signs and symptoms -Mediastinal infection -Positive blood culture
Non MCS infections: infections that do not directly arise from MCSD/components but may be related to it and can affect it.	
1. Infective endocarditis of native or prosthetic valves	Per modified Dukes criteria
2. Cardiac IED infections	Per Heart Rhythm Society Expert Consensus Statement
3. Non MCS bloodstream infection	Positive blood culture from non MCS source like UTI, pneumonia, abdominal abscess, CVC infection, etc.
4. Sternal wound infection and mediastinitis	Superficial mediastinal or thoracotomy wound infection Sternal osteomyelitis Mediastinitis
5. Sepsis	Per Surviving Sepsis Campaign 2021
6. Localized infections	Localized infections not contiguous to MCSD/components

### Infection diagnosis

#### Clinical

The infection sites (driveline, pocket, vascular cannulation) may have skin erythema and/or induration, pain/tenderness, purulent discharge, and drainage. Systemic signs and symptoms of infection may be present. However, indolent infections without overt signs and minimal drainage are not uncommon. It is important to look for pocket infections and VAD endocarditis in cases with persistent drainage or resistant infections (38).

Suspected infection should evaluate the source, type, extent, and microbiology of infection. Diagnosis relies on laboratory measures (CBC, CRP, LDH), bacterial and fungal cultures from sites of suspected infection and blood, imaging and surgical assessment (12). Identifying causative pathogens and determining device involvement are critical, as they can establish treatment duration and need for intervention for source control. Indwelling lines and catheters especially those in place >=14 days are major risk factors for nosocomial infections. Temporary non-cuffed/non-tunneled catheters have a greater risk of infection compared to PICCs and cuffed/tunneled catheters. Catheter associated urinary tract infection (CAUTI) is the most common non-VAD infection (13).

#### Imaging

Various imaging modalities, including echocardiography, ultrasonography, computed tomography (CT), and nuclear imaging, have been employed for MCS-related infection detection. Each has unique roles and limitations.

##### Echocardiography

In LVAD patients, TTE is typically the initial test for detecting valvular vegetations or, rarely, device endocarditis involving inflow regions. TEE is specifically recommended when infective endocarditis is suspected clinically and before transitioning from intravenous to oral antibiotics in endocarditis (39).

Limitations in LVAD Context: Many LVAD recipients have additional intracardiac devices such as defibrillators, pacemakers, or transcatheter valves, which may also become infected. Echocardiography in LVAD patients face significant limitations: (1) acoustic shadowing and metallic artifacts obscure visualization and lower image quality (2) vegetations <5 mm are detected in only 25% of cases by TTE, and (3) differentiation between sterile thrombus and infected vegetation can be challenging, particularly on pacemaker leads.

For LVAD driveline, pump pocket, or pump housing infections, echocardiography's role is limited primarily to excluding concurrent valve endocarditis or identifying complications. Even with TEE, up to 30% of IE cases may be normal or inconclusive (39). Therefore, although widely used as an initial assessment tool, echocardiography alone is insufficient for definitive LVAD infection diagnosis, and alternative imaging modalities are required for complete evaluation.

TTE is generally performed when LVAD infection is suspected. TEE is performed when: (1) TTE is negative but high clinical suspicion remains, (2) prosthetic valves or intracardiac devices are present, (3) Staphylococcus aureus or Enterococcus faecalis bacteremia is present, or (4) before switching from intravenous to oral antibiotic therapy in endocarditis. If both TTE and TEE are negative, but suspicion persists, repeat imaging in 3–7 days or proceed to advanced imaging modalities (39).

##### Ultrasound

Point-of-care ultrasound can evaluate superficial fluid collections at the driveline exit site and guide percutaneous drainage of accessible abscesses. Advantages include real-time visualization, portability, lack of radiation, and ability to perform at bedside. Doppler ultrasound can assess increased vascularity, suggesting inflammation. However, ultrasound has substantial limitations for LVAD infection assessment: (1) cannot reliably image deep infections beyond 5–6 cm depth, (2) acoustic shadowing from device components prevents visualization of pump pocket and driveline tunnel, (3) air in soft tissues (which may accompany infection) creates artifacts, and (4) cannot differentiate sterile from infected fluid collections. Like echocardiography, ultrasound cannot be used for definitive diagnosis or exclusion of LVAD-specific infections and should be considered complementary to more definitive imaging modalities.

##### Computed tomography (CT)

Non-contrast and contrast-enhanced CT are valuable in assessing superficial and deep infections in patients with LVADs. CT can identify early signs of infection, such as inflammatory changes, abscesses, or fluid collections. By identifying the site and extent of the infection, CT can guide appropriate management decisions including surgical planning. Peritoneal fat stranding, localized fluid collections, especially those with rim enhancement, and gas bubbles can suggest infections. However, these need to be interpreted with caution in the early postoperative setting since normal post implant CT scans can have similar findings for a few months after surgery. CT also has some value in prediction: infiltrative changes 14 mm or larger surrounding the driveline, and preperitoneal location of infection is associated with subsequent pump pocket or mediastinal abscess (40). In a retrospective study of 129 patients with LVAD infection, the absence of superficial fat stranding on CT significantly predicted infections that are unlikely to require surgical therapy (41). A major advantage of CT is its ease and widespread availability. Limitations include inability to differentiate sterile fluid collections from infection, inability to provide microbial specificity, and metal artifacts hamper image quality, a problem being addressed with metal artifact reduction algorithms.

##### 18F-Fluorodeoxyglucose positron emission tomography/computed tomography (FDG-PET/CT)

**Figure 3:**
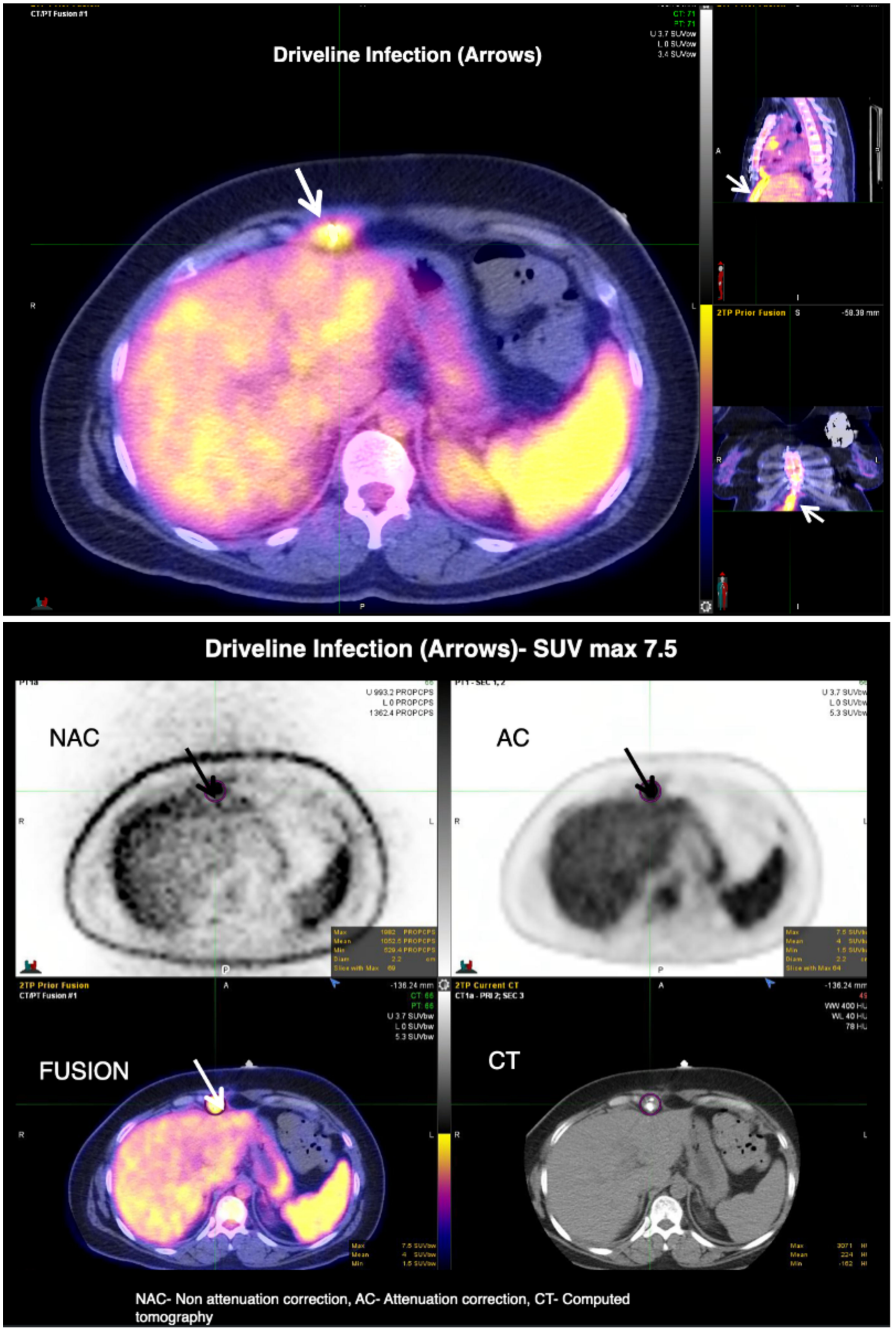
FDG-PET images of driveline infection.

**Figure 4:**
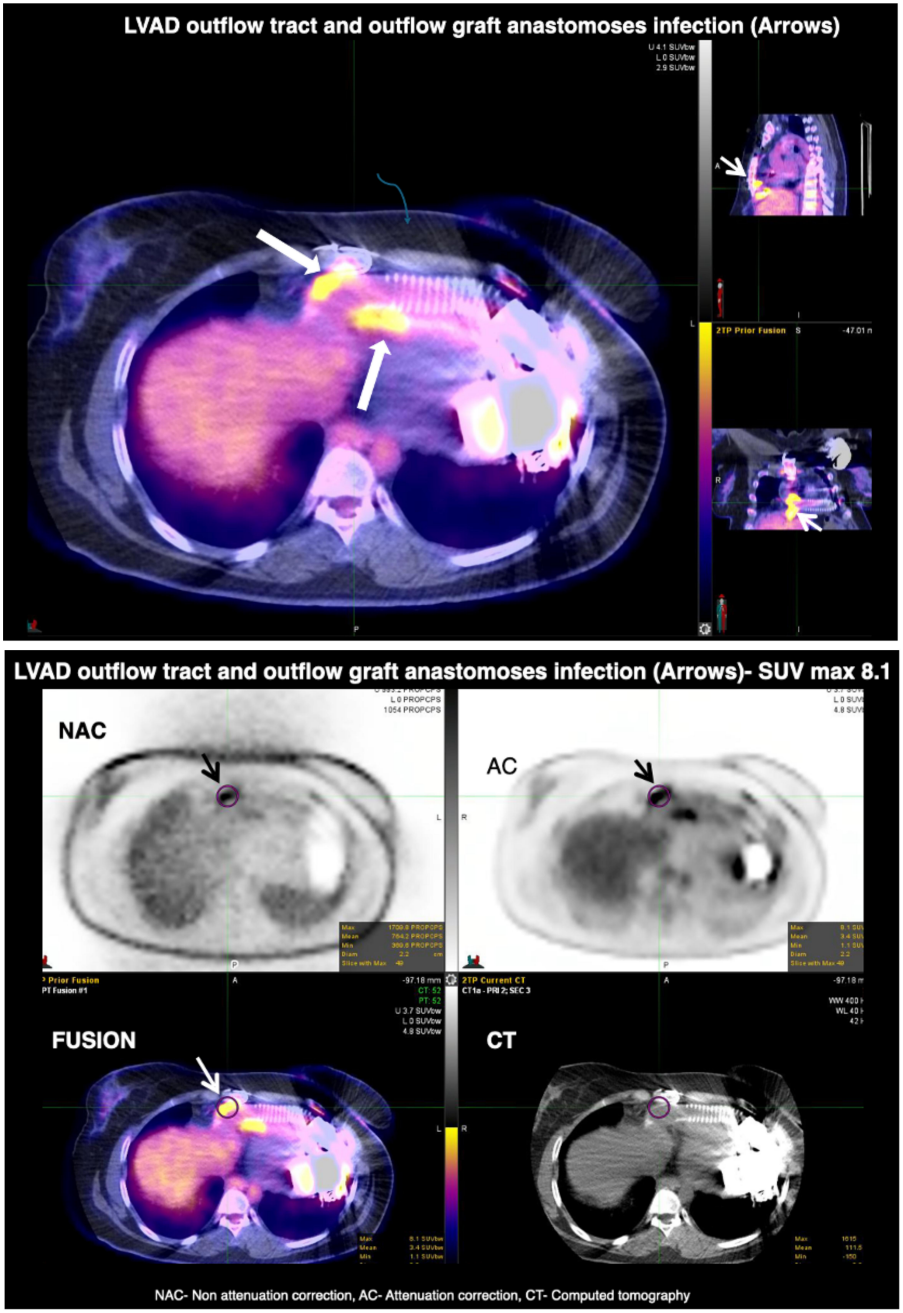
FDG-PET images of LVAD outflow tract and outflow graft anastomoses infection.

FDG-PET/CT is the most accurate noninvasive imaging modality for assessing LVAD infection, supported by a growing evidence base (Figures 3, 4).

A 2019 meta-analysis (4 studies, 119 scans) showed strong performance with 92% sensitivity, 83% specificity, and ROC AUC 0.94 (42). Additional analyses (8 studies, 230 patients) demonstrated sensitivity/specificity of 0.95/0.91 for device infection, 0.97/0.93 for pump/pocket infection, and 0.96/0.99 for driveline infection (42). SUV >2.5 may optimally identify culture-positive driveline (42–44).

In a 35-patient study, PET outperformed CT: CT alone suggested infection in 11%, whereas FDG-PET detected metabolic evidence in 80%, including central device involvement in 23 patients—findings with major management implications (45). FDG-PET diagnosed infection of 3–4 compared to fewer LVAD components, and central compared to peripheral infection was associated with worse outcomes (45, 46). PET also has management and prognostic implications: in a 2025 study of 40 patients with LVAD infection, PET affected clinical management in 83% of patients, including surgical debridement (8%), pump exchange (13%), transplant listing status upgrade (10%), and 8% urgent transplants (47). Patients undergoing early image-guided surgical intervention within 3 months after PET/CT (*n* = 21) required significantly less inpatient hospital care during follow-up compared to those receiving delayed surgical revision (*n* = 11; *p* < 0.05), suggesting early PET-guided intervention may facilitate less complicated subsequent courses.

FDG-PET/CT uniquely assesses systemic inflammation: driveline infection was associated with increased uptake in the thoracic aorta, spleen, and bone marrow in a 118-patient study (46). Parametric PET is an emerging advancement, enabling dynamic imaging and kinetic modeling. In a 23-patient prospective study, it improved lesion contrast, revealed metabolic heterogeneity, and correlated better with biomarkers (48).

PET limitations include false positives in early postoperative periods (<6–8 weeks), need for dietary preparation, limited availability, higher cost, and interpretation challenges. Current guidelines acknowledge the appropriateness and value of PET in selected cases without mandating use. The clinical role of PET is primarily when suspicion persists despite negative or equivocal echo/CT, for defining infection extent before surgery or transplant, monitoring treatment response, and detecting metastatic infection. It should complement—not replace—standard diagnostic workup.

##### Radiolabeled leukocyte scintigraphy also helps identify MCS-related infections

However, despite high specificity and positive predictive value, tagged leukocyte scans exhibit low sensitivity and negative predictive value compared to FDG-PET for diagnosing LVAD infections (49). Additionally, leukocyte imaging requires 18–24 h for cell labeling and imaging versus PET's same-day protocol, and images are relatively count-poor with inferior spatial resolution.

##### Multimodality imaging strategy

Contemporary practice increasingly emphasizes a multimodality imaging approach rather than reliance on a single modality. Initial assessment begins with TTE ± TEE for suspected LVAD infections to evaluate concurrent valve endocarditis and assess device inflow/cannula regions. Contrast-enhanced CT is performed for patients with suspected deep infection, abscess, or when surgical planning is needed. When echocardiography and CT are inconclusive but clinical suspicion remains high, FDG-PET/CT provides metabolic confirmation of infection, defines extent, reveals concealed deep infection, identifies distant septic emboli, and offers prognostic information. Each modality contributes to unique information. CT and PET can be complementary: PET can demonstrate infection not evident on CT, but CT can provide essential anatomic context for surgical planning. While multimodality imaging increases costs, appropriate sequencing—reserving PET for cases where it will change management—optimizes value. For example, early accurate diagnosis with PET may prevent futile surgeries or enable earlier appropriate intervention, which may ultimately reduce overall costs despite higher imaging expenses.

Emerging imaging techniques relevant for LVAD patients include newer tracers, including infection-specific radiotracers (e.g., radiolabeled antimicrobial peptides, pathogen-specific probes), machine learning algorithms integrating multimodality imaging data to predict infection presence, extent, and outcomes, and photoacousting imaging, which is a hybrid optical-ultrasound technique offering molecular imaging without radiation.

### Management

Management of LVAD infections depends on infection site, pathogen, and patient factors such as acuity and transplant candidacy. Empiric therapy should provide coverage for methicillin-resistant staphylococci and *Pseudomonas aeruginosa* and subsequently be tailored to culture results; however, in patients with a history of more resistant organisms, expanded coverage targeting carbapenemase-producing organisms or vancomycin-resistant organisms should be considered. Non-VAD related infections typically do not require alterations in duration of therapy, unless there is a complicating bloodstream infection. Superficial exit-site infections without systemic illness may be treated as an outpatient with culture targeted antimicrobial therapy (via oral or intravenous antibiotics) for at least two weeks, whereas other VAD-specific infection deep driveline, pocket, pump, or cannula infections, require hospitalization and intravenous therapy. Deep driveline and pump/cannula infections can require ≥6 weeks of intravenous antibiotics with adjunctive surgical management unless definitive source control is achieved. Chronic suppressive antibiotics may not reliably prevent relapse with approximately 40% of patients failing suppressive therapy; however suppressive therapy may delay time to relapse (50, 51). Side effects, warfarin interactions, and development of antibiotic resistance are considerations. Decisions for long-term or indefinite suppression are individualized and consider multiple factors including the culprit organism, its ability to create a biofilm, acuity of illness with infection, and candidacy and anticipated timing for transplant.

Candidacy for device exchange, explant, or transplantation should be assessed, with antibiotics continued through transplant. Data on antimicrobial pharmacokinetics in durable MCS are limited; dosing is standard, though altered vancomycin kinetics have been reported, supporting therapeutic drug monitoring when available (52). Because many antimicrobials interact with warfarin, close INR monitoring and anticoagulation adjustment are recommended.

#### Surgical management of LVAD infections

Surgical management of LVAD infection complements antimicrobial therapy and may include debridement, vacuum-assisted therapy, driveline repositioning, or flap coverage (53). Refractory cases may require LVAD exchange or heart transplantation as a last resort (54–56). Surgical intervention is indicated for recurrent or persistent driveline infections, deep tissue or pocket involvement, abscess or fistula formation, device-related sepsis, or necrotizing infection. Decisions are guided by infection extent, systemic involvement, patient frailty and LVAD dependence, and microbiologic resistance patterns. It is important to note that evidence for surgical therapy is limited; published studies often reflect single-center approaches and experience, with potential for outcome heterogeneity. Comparative effectiveness studies are generally lacking. Management is therefore highly individualized.

##### Driveline site debridement and washout

For superficial driveline infections not near the LVAD, initial management involves surgical debridement of infected tissue and removal of velour. The infected cavity is opened as needed, avoiding contamination of the pump. Abscesses or purulent material are irrigated, optionally with antibiotics, followed by NPWT or VAC placement. Select cases may use antibiotic beads (e.g., calcium sulfate with vancomycin or tobramycin) (57). Multiple procedures may be required. Once cultures are negative, the driveline can be tunneled under the rectus muscle to enhance healing and reduce infection risk.

##### Negative pressure wound therapy (NPWT)

Several small case reports and series have shown a potential role for adjunctive NPWT in DI (58, 59). Another series of 3 patients with infected and exposed LVADs showed favorable results with NPWT with Dakin's 0.125% solution instillation, without the need for device removal (60). NPWT can be used for superficial or deep infections with persistent drainage, poor granulation, failure of standard dressing changes. It can also be used as an adjunct to surgical debridement, potentially in conjunction with instillation therapy with antiseptic solution before definitive closure with or without omental flaps. Caution is advised if there are exposed blood vessels, anastomotic sites, organs, or nerves without adequate coverage, if there is necrotic tissue with eschar not debrided, significant bleeding diatheses, proximity to driveline cable, outflow graft, or pump.

##### Driveline relocation/ reposition

The skin incision is made above the run of the driveline, and the driveline velour is removed from the driveline. The former driveline site may be treated with vacuum dressing and secondary suture closure, or with primary would closure in proximal infections. A new incision is made on the contralateral side of the abdomen, creating a new driveline. Another technique of re-tunneling of the driveline utilizing a sterile piece of plastic tubing that was tunneled to a new exit site has been reported as well. The skin over the new driveline placement is then sutured (53).

Driveline relocation is more invasive than standard I&D, yet infection remains challenging. Recurrent infection rates were similar after I&D and relocation in one series (47%), with mean time to reinfection of 135 vs. 261 days, respectively (*p* = 0.86) (61). Advantages include preserving the LVAD and avoiding device exchange. Relocation—embedding the driveline within rectus muscle fibers beneath the fascia—reduces bacterial load and, when combined with VAC therapy until cultures are negative, was successful in 90% of patients in one series (62). However, it does not fully eliminate reinfection risk.

##### Flap coverage of driveline (omentum)

The omentum's vascularity, flexibility, and immunologic properties aid infection control and wound healing. It has been used in LVAD infections, alongside driveline debridement, relocation, or pump exchange for refractory cases (54, 63). In a series of six patients with infection near the pump, laparoscopically harvested omental flaps wrapped around the driveline at a median of 3.6 years post-LVAD prevented recurrence over 2.4 months of chronic antibiotic therapy (64).

##### LVAD pump exchange

This approach offers more definitive infection control than driveline debridement and is reserved for severe, antibiotic-refractory cases (65, 66). However, the outcomes of such interventions, namely successful eradication of infection and mortality benefit after LVAD exchange, are not well understood. A 2018 meta-analysis of 158 cases found no significant differences in overall mortality or infection recurrence rate long-term with device exchange vs. nonexchange modalities of treatment (67). Infection recurrence rate post LVAD exchange was still quite high at around 27% at mean follow up of 290 days (55). In a single-center study of 49 patients, 1-year freedom from recurrent DI was 76%, with longer time from diagnosis to exchange reducing success (68). Perioperative mortality after LVAD pump exchange is approximately 5%. Device exchange is recommended for refractory infections involving the pump or pocket when other options, like imminent transplantation, are unavailable, but it entails high patient surgical stress.

##### Heart transplant

Heart transplant can eliminate LVAD-related infections. The caveat is that the patient may not be eligible for heart transplant if the infection is not adequately controlled. LVAD infection by itself is not a contraindication to transplantation as long as there is no sepsis or hemodynamic instability, organism-specific therapy with confirmed susceptibility is available, and bacteremia is ideally resolved. Caution is advised with invasive mold infections given high post-transplant mortality. It is also important evaluate for persistent bacteremia and for metastatic seeding that can be associated with LVAD infections. Diagnosis can be challenging; a combination of blood or tissue cultures, standard imaging such as CT, and advanced imaging with PET may be required if there is concern for metastatic infection. Pre-transplant source control, including drainage of any abscesses, debridement of any necrotic tissue, and resection of any mycotic aneurysms may be necessary. Any potentially infected foreign material such as pacemaker and defibrillator leads or driveline fragments are removed at the time of transplant. Pathogen-specific therapy is continued post-transplant in consultation with infectious diseases specialist.

In appropriately selected patients transplanted with LVAD infections, survival is similar to other transplant populations (56, 69). LVAD infection is a risk factor for post-transplant infection (70). A meta-analysis of 13 studies (>6000 patients) found pre-LVAD infection associated with 30% higher post-transplant mortality, though differences in device type, era, transplantation practices, specifics of LVAD infection and severity, as well as the inability to distinguish whether transplantation was performed with incidental DI history versus with specific goal of refractory LVAD infection treatment limit applicability to current practice (71).

In summary, surgical management of LVAD infections depends on the severity and depth of the infection, and the patient's overall health status. Driveline site debridement and relocation are good options to control local infection. However, if the infection is deeper, pump pocket debridement or LVAD exchange may be necessary. If the infection is refractory to these treatment options, heart transplantation remains the definitive option.

### Outcomes

Infections in LVAD patients remain a major cause of morbidity and mortality, with over 50% of patients experiencing major infection by 2 years. LVAD infections lead to hospital admissions and readmissions, long hospital lengths of stay, increased predisposition to other complications such as stroke, and increased mortality (72). In an INTERMACS analysis, compared to patients without infection, those who had a VAD-related or VAD-specific first infection had a three-fold hazard of mortality (73). In the ISHLT Mechanically Assisted Circulatory Support registry, survival at 24 months following infection was 59% compared to 74.8% for those without infection. Higher 12-month survival was observed in those with driveline infection (86.4%) compared to those with pump/cannula infections (58.5%) or VAD pocket infections (66%) (8). Beyond survival, chronic infections reduce quality of life through repeated interventions, prolonged antibiotics, pain, and caregiver burden.

### Novel therapies and future directions

Implanted devices with transcutaneous energy transfer and elimination of the driveline have been developed for many years. Infection rates were lower, but other challenges precluded widespread use (74). Recent advances in wireless and coplanar energy transfer, as well as smaller, more flexible drivelines (e.g., BrioVAD), may further reduce infection risk (75, 76).

Novel infection-specific PET tracers such as ^11^C-Trimethoprim, ^11^C para-Aminobenzoic acid, which are designed to differentiate between aseptic inflammation and true infection, are in development. Newer antibiotics such as albavancin, oritavancin, and delafloxacin may have a role in complicated LVAD infections (77). Cold atmospheric plasma which can generate bactericidal reactive oxygen and nitrogen species is being evaluated for driveline infections (78). Immune modulation as a strategy for LVAD infection management is also an approach under consideration.

Biofilms remain a major challenge in LVAD infections. Emerging strategies include nanoparticles that disrupt biofilms through multiple mechanisms, including antimicrobial delivery and CRISPR-Cas9 targeting (79, 80), and 3D scaffold for controlled antimicrobial delivery.

Phage therapy refers to the use of bacterial viruses to target biofilm-related bacteria. Personalization of phages to the bacterial strains affecting individual patients and customization of phages is possible (81). Limited data is available in LVAD patients with varying reports of success (82, 83). Further investigation and refinement are necessary, but phage therapy may emerge as a personalized therapy for LVAD infections.

### Summary

LVAD infections, affecting device and non-device components, increase morbidity, mortality, and hospitalizations, and can affect transplant eligibility. Risk factors include patient comorbidities, device type, surgical technique, and post-implant care. Management ranges from antibiotics to surgical intervention, including transplant, with emerging strategies showing promise (Table 3).

**Table 3:** Clinical framework.

Infections in mechanical circulatory support devices: Clinical framework		
Prevention	Diagnosis	Management
Pre-implant Defer implant until any infection adequately treated: source control achieved and blood cultures negative MRSA nasal screen-decolonize if positive Dental clearance Optimize glycemia, nutrition, vaccinations	Classification (ISHLT 2024) MCS-specific: percutaneous lead/driveline (complicated vs uncomplicated), vascular cannulation site, device BSI, device endocarditis, external surface infection Non-MCS: native/prosthetic valve endocarditis, cardiac IED infection, non-device BSI, sternal wound, sepsis, localized infection Clinical assessment Inspect exit site, pocket, cannula sites for erythema, induration, drainage Systemic signs may be absent in indolent infections Labs to include exit site and blood cultures WBC, CRP may be nonspecific in critical illness ECMO and CRRT may mask fever Key Microbiology *S. aureus*, *S. epidermidis:* predominant early pathogens; biofilm-forming *Pseudomonas aeruginosa:* most common gram-negative; high treatment failure Candida species: <10% but ∼25% mortality; consider in high-risk/prolonged ECMO Imaging Exit site US to evaluate for superficial fluid collections amenable to drainage TTE to screen for endocarditis TEE: if bacteremia with inconclusive TTE, concern for endocarditis, LVAD pump infection, ICD lead infection CT with contrast if suspected deep infection, abscess, or surgical planning needed FDG-PET: if echo + CT inconclusive; useful for defining extent before surgery or transplant; assessing treatment response	Antimicrobial Principles Empiric coverage: *Staphylococci* + *P. aeruginosa*; tailor to sensitivities and local antibiogram Superficial exit-site infection without systemic signs: oral or IV ≥2 wk; outpatient or inpatient Deep driveline/ pocket/ pump/ cannula or systemic infection: IV ≥6 wk + adjunctive surgical + long-term suppression unless definitive source control INR surveillance for warfarin interactions ECMO: involve ICU pharmacist for drug-specific dosing adjustments Continue antibiotics through heart transplant in patients with active LVAD infection
Perioperative Prophylactic Antibiotics 24–48h Impregnate driveline velour per manufacturer recommendations Bury velour fully, silicone exit interface C-loop tunneling or double tunnelling Consider negative pressure wound therapy (NPWT) post implant		Surgical therapies for durable MCS Debridement + NPWT: superficial DI without proximity to pump; irrigation ± antibiotic beads Driveline relocation: refractory local DI; new exit site on contralateral abdomen; embed under rectus fascia; Omental flap— refractory cases near pump; laparoscopic harvest; leverages vascularity and immunologic properties LVAD pump exchange: reserved for severe antibiotic-refractory pump/pocket infection; ∼27% reinfection rate; perioperative mortality ∼5% Heart transplant:definitive option; requires adequate infection control; outcomes comparable to non-infected LVAD if appropriately selected
Post-implant Aseptic dressing-initially daily, taper to 1–3x week once healed Chlorhexidine-based antiseptics Consider silver dressing Driveline anchor limiting motion at exit site Structured driveline management protocols		
Temporary MCS-specific Minimize support duration, plan exit strategy early Avoid MCS lines for routine labs HOB elevation, early enteral feeding, early tracheostomy if prolonged intubation Restrictive transfusion strategy		Temporary MCS-specific Device removal effective, not always feasible: indicated for severe sepsis, bacteremia >3 days, septic emboli *S. aureus*, *Pseudomonas*, fungi: high resistance to medical therapy; prioritize device removal/exchange Device de-escalation or site switch (e.g., femoral VA-ECMO → axillary Impella 5.5) may reduce infection while maintaining hemodynamic support.

## Acute MCS infections

### Types, design, and mechanics

Temporary mechanical circulatory support (tMCS) devices provide short-term hemodynamic support to maintain systemic perfusion with an intended exit strategy of recovery, durable support, or transplantation (84).

They are classified by degree of support, ventricle supported, implantation approach, mechanism, and anticipated duration. Devices range from smaller catheter-based systems such as IABP and microaxial pumps (Impella) to extracorporeal platforms with large-bore cannulation, including VA ECMO, and may be combined in hybrid configurations (85, 86).

Compared to durable MCS devices, tMCS are inherently time-limited and generally require less invasive surgical implantation. Many devices, such as IABP, Impella CP, or ECMO can be percutaneously instituted whereas others such as Impella 5.5 require surgical implantation. tMCS are more often placed in more emergent situations than durable MCS devices in more critically ill patients, which may impact infection risk.

Despite their clinical utility, tMCS devices require intensive care and are associated with significant complications, with infection remaining a major contributor to morbidity and mortality.

### Definitions

Single center studies, registries, and the few randomized controlled trials of tMCS devices have all used varying infection definitions, limiting comparability between studies. In 2024, the ISHLT consensus statement attempted to harmonize definitions, and include both acute and durable devices (Table 2). Infections in MCS patients include device-specific and non-device-specific infections, which can include cardiac, septic, or localized infections (e.g., UTI, pneumonia) (87).

### Epidemiology

Data on infections in temporary MCS (tMCS) are limited, often from small, single-center, retrospective studies with variable definitions and follow-up.

Intra-aortic balloon pump (IABP) remains the most widely available MCS device worldwide. In 403 patients with IABP, the infection rate was 7.2% (88). Axillary IABP may be performed to allow ambulation, and sometimes for longer duration compared to femoral IABP when used as bridge to transplant. In 141 axillary IABP patients, bloodstream infection (BSI) incidence was 13.1%. Patients on prophylactic antibiotics had reduced BSI (24% vs. 8%; *p* = 0.01) (89). Patients who require multiple MCS devices or device escalation have higher infection rates (90). In the IABP-SHOCK II trial of IABP vs. medical therapy in acute MI-cardiogenic shock, 15.7% in the device arm had sepsis in the hospital, but 20.5% in the control arm had sepsis, underscoring the point that it is the critically ill patient, not just the device that is important in infection (91).

In a single-center study of 249 patients with cardiogenic shock on Impella or VA ECMO, 20% developed BSI after MCS placement, at a median CICU length of stay of 13 days. Twenty percent of organisms were drug resistant. No difference in unadjusted 30-day mortality was found between those with and without infections (92). In the randomized DANGER-SHOCK, 11.7% in the Impella arm had sepsis with bacteremia (93).

Heart transplant candidates are increasingly bridged to transplant (BTT) with tMCS devices. In 249 BTT tMCS patients from 16 centers, 34.9% developed infections—mostly respiratory (46.1%) or urinary (15.6%). MCS-specific infections were rare (3.4%), while MCS-related were 23.6% and non-MCS 73%. Gram-negative bacteria predominated (58%). Longer support increased infection risk. Infection was associated with higher mortality (25.3% vs. 12.3%) and lower transplant likelihood (73.6% vs. 85.2%). Rates of infections in surgical VADs, percutaneous VAD, central ECMO, and peripheral ECMO were 50.6%, 27.3%, 40%, and 26% (*p* 0.002) (94). In 444 axillary Impella 5.5 patients (median support 21 days), bacteremia occurred in 5%, any infection in 16.7% (95).

Patients on ECMO reflect a high-acuity patient cohort with significant infection burden (87, 96). In 220 VA ECMO patients, nosocomial infection rate was 75.5 episodes per 1000 ECMO days, with time to first nosocomial infection 8 days on support. Pseudomonas aeruginosa was the most common organism identified from respiratory or bloodstream samples. The admission SOFA score was independently associated with nosocomial infection. Nosocomial infection with severe sepsis/shock predicted mortality, and 27 deaths were directly attributable to infections (97). Other cohorts have reported infection rates ranging from 23% to 42%, most commonly pulmonary (98). In another single-center cohort of 69 patients on VA ECMO, 42% developed infection, most commonly pulmonary. The attributable mortality from blood stream infections and ventilator associated pneumonia was 13% (99). Ventilator associated pneumonia (VAP) was an important cause of mortality (HR 3.05) in VA ECMO patients, and need for renal replacement therapy and infection with pseudomonas aeruginosa were risk factors for treatment failure in VA ECMO patients with VAP (100).

### Preventive measures

Risk factors associated with infections in tMCS include duration of mechanical support, hospital duration, underlying disease and severity, mechanical ventilation and hemodialysis, and host factors including age and immunosuppression (87, 101). Early identification of high-risk patients and preventive strategies—shorter support duration, careful cannulation, hand hygiene, circuit sterility, regular monitoring, and timely treatment—are essential. Device-specific training and competency assessment for staff are recommended. Chlorhexidine is recommended for disinfection.

Other measures include avoiding MCS lines for routine blood draws, preventing pressure injuries, elevating the head of the bed, early enteral feeding, and limiting invasive devices (84). Prolonged ECMO (>250 h) increases BSI risk, while prolonged intubation raises VAP risk; early tracheostomy reduces infections and improves outcomes (101). Given transfusion-related immunomodulation by various mechanisms including allogeneic leukocyte-mediated immunosuppression, accumulation of bioactive substances in blood, and impaired NK cell, neutrophil, and macrophage function, restrictive transfusion strategies (goal Hb ≥7 g/dL) are suggested to minimize immunosuppression-related infection risk (102, 103).

### Diagnosis

#### Clinical

Frequent monitoring for signs of infection, scheduled inspection of cannulation sites, and transparent dressing when possible are some measures for early diagnosis. It may be challenging to detect infection: critically ill cardiogenic shock patients may have systemic inflammatory response syndrome that may physiologically and clinically mimic sepsis. WBC, CRP, and procalcitonin are nonspecific in acutely ill patients. In patients on ECMO or CRRT, temperature control via the device may mask fever. Drainage samples for bacterial and fungal culture, and cultures of explanted device or cannula are also helpful in diagnosis and isolation of organisms. Blood cultures may be positive during or within 48 h of device removal (84). Standard diagnostic criteria have been recommended for diagnosis of non MCS specific infections (1).

#### Imaging

Patients on tMCS often require additional life support and are at a heightened risk of nosocomial infections from critical care needs like intubation, ventilator support, and intravascular or urinary catheters. Imaging sites and modalities may be tailored to sites based on clinical suspicion (104). TTE has low sensitivity for device or native endocarditis. TEE may be helpful, but image quality is decreased by artifacts from metallic devices. Differentiating between sterile and infected vegetation or clots, particularly attached to intravascular lines or pacemaker leads, can be challenging, and nuclear imaging may be considered for further evaluation.

### Management

#### Medical therapy

There are no standardized guidelines for prophylactic antibiotics in all tMCS. The ELSO Infectious Disease Task Force recommends standard surgical prophylaxis (105).

Antibiotic prophylaxis is recommended for centrally cannulated devices and peripheral devices placed via surgical cutdown, typically with cefazolin or vancomycin. For infection treatment, choice of therapy should consider patient factors, immune status, and procedural sterility, with broad-spectrum coverage and antifungal therapy in high-risk patients (84). Percutaneous device infections should be managed like central line infections, while surgically implanted devices should follow surgical site infection protocols. In contrast to durable MCS devices, infections associated with temporary mechanical circulatory support can often be managed with pathogen-directed intravenous antimicrobial therapy and device removal when hemodynamically feasible, obviating the need for prolonged suppressive antibiotic regimens.

#### Pharmacokinetic and pharmacodynamics on ECMO

In ECMO, pharmacokinetic (PK) and pharmacodynamic(PD) parameters are influenced by the drug, critical illness disease process, and the presence of extracorporeal factors. ECMO alters volume of distribution (Vd) and drug clearance (106).

ECMO patients often have reduced clearance due to renal and hepatic hypoperfusion. Hydrophilic drugs have low Vd but may be diluted by priming solutions, lowering plasma levels, while lipophilic drugs may sequester in tubing and oxygenators, increasing Vd. Diluted plasma proteins can raise free fractions of highly protein-bound drugs, increasing toxicity risk (106). ECMO patients may have lower drug clearances related to renal and hepatic hypoperfusion and hypoxia (107). These changes complicate antimicrobial dosing and make dosing nomograms unreliable, requiring drug-specific adjustments and ICU pharmacist collaboration (108). Therapeutic drug monitoring is important to confirm that antibiotics meet pathogen-specific PK/PD targets while avoiding toxicity in patients with compromised renal and hepatic function, particularly when pharmacokinetic parameters are shifting as device flows, fluid balance, and organ function evolve.

#### Device removal, exchange, or weaning

A major difference between tMCS and durable MCS infection management is the easier potential for device removal. Device removal is the most effective way to eradicate infection but may not be feasible due to hemodynamic dependence. Device removal is generally indicated in the setting of severe sepsis, persistent bacteremia for >3 days or evidence of septic emboli. Infection with Staphylococcus aureus, pseudomonas, non-tuberculous mycobacteria, and fungi are often resistant to medical therapy (84). If device/cannula is changed, tissue, fluid, and other surgical samples can be collected for targeted therapy (1). In some cases, device de-escalation, or site switch (e.g., femoral VA ECMO to axillary Impella 5.5) may reduce infection while providing hemodynamic support.

## Conclusion

Infections are important complications in patients on tMCS devices. As the use of tMCS continues to grow, continued investigation into device-specific risks, diagnostic challenges, and targeted therapies is needed to improve outcomes.
